# Where are my Black professors? The research culture preventing Black researchers from thriving in UK institutions

**DOI:** 10.12688/wellcomeopenres.24584.1

**Published:** 2025-09-04

**Authors:** Sarah Essilfie-Quaye, Sasiragha Priscilla Reddy, Mary Adeturinmo, Clare M Lloyd, Winston Morgan

**Affiliations:** 1 Faculty of Medicine, Imperial College London, London, England, SW7 2AZ, UK; 2 Centre for Health Economics & Policy Innovation, Imperial Business School, London, England, SW7 2AZ, UK; 3 National Heart and Lung Institute, Imperial College London, London, England, SW7 2AZ, UK; 4 Department of Bioscience, School of Health Sport and Bioscience, University of East London, London, England, E15 4LZ, UK

**Keywords:** Research culture, Black heritage, equity, biomedical research, Black researchers, Black academics, #BlackintheIvory, #BlackinSTEM

## Abstract

This paper investigates the persistent underrepresentation of Black heritage researchers and academics at UK Higher Education Institutions in Science, Technology, Engineering, Maths, and Medicine (STEMM), with a focus on Biomedical Research where possible. Despite decades of policy development, research, and advocacy, 0.6% of STEMM professors are Black, falling to zero in certain disciplines. Are the structural barriers that exclude Black researchers at every stage of the STEMM pipeline?

Research culture influences how research is conducted, communicated, and how researchers are supported. Academic progression depends on research, funding, publications, teaching, and leadership, and the criteria are often unequally applied. Evidence highlights Black researchers’ experiences of exclusion, racial harassment, and opaque advancement processes; moreover, funding disparities further entrench inequality. Data reveal that Black researchers account for less than 1% of fellowship holders and Principal Investigators. Additionally, without institutional support, Black researchers and academics are routinely excluded from the research culture and economy.

Black professors’ underrepresentation is not simply a pipeline issue but a result of intersecting failures in recruitment, funding, and academic culture. Grassroots initiatives offer community support but come at a cost, the “Minority Tax”, which is unpaid labour that diverts time from career-building activities.

Representation matters for students, knowledge production, and institutional legitimacy. Therefore, systemic reforms are urgently required. Research funders and institutions must implement transparent, equitable policies that enable Black researchers to not only enter but also thrive and lead within the UK research ecosystem.

## Plain Language Summary

Black heritage researchers are significantly underrepresented in science, technology, engineering, mathematics, and medicine (STEMM) in UK institutions. Four percent of the population comes from a Black background; however, despite years of policies and efforts to improve diversity, less than one percent of STEMM professors are Black, and in some fields, there are none.

This study examined the reasons for this ongoing inequality. Black researchers face structural barriers at every stage of their science journeys from education to leadership, and the way research is selected, funded, and supported often hinders and acts against them. Black academics frequently report feeling excluded, experiencing racism, or being left out of important career opportunities. They are also less likely to receive research funding or to hold senior roles.

The answer is not just about getting more Black heritage people into the system. It concerns the research culture of the system and how this affects both the researchers and the people in charge of making decisions within this system. This under-representation is important. Diverse voices improve research quality, support students, and strengthen institutions.

This paper calls for urgent, system-wide changes; ultimately, the people responsible for funding research or leading our institutions should ensure that there are fair and equal opportunities for Black researchers to succeed and lead in UK research.

## Introduction

### Ethnic diversity in the UK and inequalities

Just over 18% of the population (England and Wales) are from Black (4.0%), Asian (9.3%), Mixed Race (2.9%), or other ethnically diverse backgrounds (2.1%) ^1^. Discrimination and inequalities affecting these minoritised ethnic groups in housing, criminal justice, economic outcomes, education, and health have been documented and reported since a large number of people were invited to the UK from other Commonwealth countries after the end of the Second World War ^2^. This led to the promulgation of several UK Race Relations Acts, the first of which was passed in 1965 ^3^, followed by a series of legislations with at least six editions of the Act between 1965 and 2000, culminating in the 2010 Equality Act ^4^. Despite these acts, numerous reports and studies have confirmed that discrimination and inequalities persist and that the picture in terms of causes and resolution is complex ^5, 6^. One area for which there are extensive inequalities linked to race and ethnicity is health outcomes, despite the vast sums being spent annually by the NHS on treatment and by the research council’s awards to universities for biomedical research. A key factor sustaining inequalities in health outcomes could be the paucity of Black and other minoritised groups involved in biomedical research at our key institutions, particularly senior positions ^7, 8^. These individuals could alter not only what we research but also provide new insights into how the data generated are interpreted.

### The impact of biomedical research on health equity in the UK

Inequity in biomedical research can directly impact inequality in several areas of life. An in-depth understanding of these inequities is therefore essential for the development and application of health and healthcare interventions ^9– 11^. For the purposes of this paper, the term biomedical researchers includes clinical and academic scientists researching in Higher Education Institutions. Biomedical researchers form a large proportion of the Higher Education sector, and more importantly, their work is considered to be some of the most impactful studies being undertaken today ^12^. One of the most urgent areas for this type of research is around health inequalities, where there continues to be huge disparities in health outcomes affecting ethnically minoritised groups in the UK and elsewhere ^13– 16^. For example, the level of infant mortality among minoritised ethnic groups is two to three times higher than their White British counterparts ^17^, and Black women have maternal mortality rates two times higher than White women ^18, 19^, a figure that has only recently fallen from five times the maternal mortality rate of White women, and a steady decrease since records began in 2000 when the maternal mortality rate for Black women was seven times higher than for White women ^20^. Another example of health inequalities is the Covid-19 pandemic, which had a disproportionately larger impact on most ethnic minority communities than on White British people ^21, 22^. One possible cause of this disparity is that pulse oximetry, which measures blood oxygen levels, has not been sufficiently researched on different skin tones, leading to misdiagnosis and worse outcomes in individuals with higher levels of skin melanin ^23, 24^. Other examples of health inequalities include cardiovascular diseases, diabetes, and mental health ^5, 15, 25^. There is sufficient evidence in the literature that demonstrates that reducing inequalities in the research community could lead to a reduction in inequalities in several sectors, including health, and to a more equitable society in general. This was a key recommendation from the report produced by Professor Kevin Fenton for Public Health England, “Beyond the data” ^26^. Building on the inequalities discussed above, a similar pattern can be observed in the recruitment and training of the Science, Technology, Engineering, Maths, and Medicine (STEMM) workforce, including researchers, academics, and the broader professional cohort.

## Discussion of the recent literature

### Reviewing the landscape

By reviewing a variety of materials, including academic articles, published reports, opinion pieces in scientific journals, grey literature, government representations, and documents, it became clear that there are a limited number of instruments for quantitative measurements of issues related to inequalities in biomedical researcher careers based on ethnicity. Many studies on this topic are based on clinical or healthcare professions ^27^, student perspectives ^28^, or the USA experience ^29^. A review on the experiences of Black and minority ethnic (BME) staff in the UK Higher Education sector ^30^, and the seminal book “The Experiences of Black and Minority Ethnic Academics: a comparative study of the unequal academy” ^31^, drew on research conducted in the UK and USA. A recent editorial highlighted the need for a deeper analysis of the path to dismantle racial inequalities in Higher Education ^32^; these works do not concentrate on Black Heritage individuals. The Staying Power report, which describes the career experiences and strategies of UK Black female professors ^33^, is the first known UK study to focus exclusively on the career experiences of Black female professors. There have also been newspaper articles that played an advocacy role in raising issues and drawing attention to published reports on the current situation of Black researchers and academics in the UK ^8, 34– 36^. However, the data on professors, although important, only highlighted the impact at the highest level. In all these institutions, there is a much larger group of Black academics and researchers working at lower levels, who are being significantly affected but whose story is not being properly told ^32, 37– 39^. This underrepresentation has a double knock-on effect on minoritised students who never see a career path in research as something open to them, and where established academics find it difficult to accept that someone who does not look like them can be a successful researcher. At this stage, it is important to accept that the biggest inequalities can be seen affecting people racialised as Black as patients, researchers, and students; consequently, much of the focus and analysis will concentrate on this group ^38, 40– 43^.

### Identifying themes in the current landscape

The paucity of information creates an opportunity to conduct qualitative research to gain an understanding of inequalities in biomedical research careers and develop validated variables for future quantitative studies. Therefore, progressing this field of study further.

A literature search was conducted on inequalities in research careers using the following keywords: Black academic inequalities, structural racism, percentage of Black academics in UK institutions, racism in UK health research, and inequalities experienced among researchers.

Additionally, the search examined articles on how to measure inequality using the following themes:

- measures of inequality
- measures of hopelessness
- measures of discrimination
- measures of structural inequality

While a limited number of papers identified themes directly related to the topic, to make sense of the literature, gain an understanding of this landscape, and find relevant and informative information, the resources were categorised into themes qualitatively and further explored below. Other important themes included self-care and coping strategies for survival, racism in research dissemination, and racism in the assessment of research excellence. Recommendations and methods were explored directly and indirectly in this study.

The themes below are explored in more detail:

- Profile and representation of Black biomedical researchers in academia: demographics and social epidemiology
- Absence of critical mass of Black researchers
- Culture of biomedical research and academic education and training
- Recruitment, Promotion and progression

### Profile and representation of Black biomedical researchers in academia: Demographics and social epidemiology

A review of the literature begins to provide an understanding of inequalities among the healthcare workforce ^14, 44^, and postgraduate and undergraduate students ^45^. However, there are a limited number of studies (both qualitative and quantitative) on the academic and research workforce ^7, 46^. The research workforce can be defined as those working on basic research, applied research, or experimental development ^47, 48^.

The Stuart Hall Foundation, in partnership with the Centre on the Dynamics of Ethnicity, prepared a comprehensive review ^6^ of 13 reports spanning 37 years, from 1981 to 2017, investigating inequalities that affect minoritised ethnic groups in the UK. This review provides useful insights into inequalities in education, employment, health, housing, policing and the criminal justice system, and community cohesion, but not academic research. The more recent Commission on Race and Ethnic Disparities report ^5^ called for more in-depth research into the root causes of health disparities in physical and mental health and touched upon the need for fairness with regard to employment. While these findings highlight persistent inequalities across the broader workforce, they also underscore the need to examine how such disparities manifest in Higher Education, given its critical role in shaping the future STEMM workforce and academic leadership *.*

### The consequence of diversity in Higher Education and the absence of critical mass of Black researchers

The business case outlining the advantages of diversity in various sectors has been widely reported and accepted, and examples are increasing ^49, 50^. In addition to diversity being important to the overall well-being of society ^51^, research teams that have diverse makeup of people have been shown to produce high-quality papers ^52^, and companies that have a diverse workforce and leadership teams outperform their peers in terms of profitability ^50^.

Elite universities that rank highly in global surveys are characterised by a high percentage of international postgraduate students, often exceeding 50%, suggesting that diversity is a powerful factor in increasing the excellence of an elite university ^53– 55^. Much of the biomedical research conducted in the UK is within the Higher Education sector, so it is important to understand the diversity of researchers and leadership in this sector from the beginning of people’s journey studying science at school, through to choosing a degree in this field at university ^56, 57^. Because of the limited number of Black people becoming full professors, it is important to ensure that the lived experiences of minoritised people across STEMM are sufficiently recorded and understood so that interventions can be made to correct such inequalities, and the effects of such interventions are monitored ^58^.

The latest Higher Education Statistics Agency (HESA) data ^59^ show that of the academic staff of known ethnicity, 24% were from ethnically minoritised backgrounds, a 2% increase on the previous year, which is higher than the numbers based in England and Wales (ONS 2021). The figure for the known ethnicity of professors from ethnically minoritised groups drops to 14%. Regardless of the overall figures for “minority ethnic” groups, there is a significant underrepresentation of Black academics and researchers in Higher Education (less than the 4% of the population). In the case of professors less than 1%, this confirms the greater inequity for Black staff.

### Culture of biomedical research, academic education, training and the lack of Black professors

The HESA data tells us that there are 25,380 professors in the UK, of which 17,480 are male and 8,150 are female. However, of over 25,000 professors, only 250 were recorded as being from Black heritage backgrounds. This is less than 1% of professors. Furthermore, this low figure follows a recent increase of 40 Black professors over a 12-month period, compared to the 2022/23 data ^60^.

In a panel discussion organised by University College London in 2014, UCL Talks, the question was asked: ‘Why isn’t my professor Black?’ ^61^. Over a decade later, the question remains the same, as the number of Black professors remains low.

It is therefore not surprising to see similarly low numbers of Black professors reflected in my own institution, Imperial College London, where 17% of academics are professors; yet, we currently have fewer than five recorded Black professors out of a total of 755 ^59, 62^.

This limited number of Black professors continues to be on the educational agenda and has been recognised as an area where recruitment and retention require improvement and acceleration ^61, 63, 64^.

### What progress have we seen to date?

A serious attempt was made in 2019, just before the pandemic, in the form of The Hepi Report ^65^, “The white elephant in the room: ideas for reducing racial inequalities in higher education”. The report provided a list of specific policy recommendations, including: All Higher Education Institutions should participate in the Race Equality Charter; Do groundwork to facilitate conversations about race within institutions; Make sure that work done by BME staff and students to tackle racial inequalities is recognised and rewarded; Academic faculties should look to their curricula and to other ways of addressing inequalities in their subject, such as studentships for BME candidates; Diversity practitioners within institutions need senior management diversity champions to rely upon; Avoid well-meaning but vague actions which are unlikely to effect change.

The policy recommendations were a strong proposal for what should have been put in place to implement change in Higher Education Institutions. Still, there is the question of when this will be translated into an increased number of Black researchers and ultimately Black professors.

Established in 2015, the Race Equality Charter is a voluntary accreditation scheme for UK Higher Education Institutions ^66^ *.* It aims to address and eliminate institutional and cultural barriers that impact the representation, progression, and success of minority ethnic staff and students by accrediting institutions that detail their commitment to developing and implementing initiatives to address racial inequality. The Race Equality Charter was designed to replicate the success of the Athena Swan scheme ^67^; however, unlike the Athena Swan, which had both *carrot and stick* incentives, such as grant awarding bodies making an Athena Swan award a requirement for applications ^68^, there are no such requirements for the Race Equality Charter which is a weakness.

More specifically, regarding Black professors in STEMM, in 2016, when there were 8,300 professors in science, engineering, and technology, it was reported that there were only 35 Black professors (Morgan 2016). Nine years later, how did the situation change? The latest HESA data state that out of 520 chemistry professors and 825 in physics, there are no Black professors, zero *.* However, since 2008, Professor Robert Mokaya has specifically and repeatedly called out that he does indeed exist; despite being reported as zero, he is actually the one Black Chemistry Professor, because data are rounded to the nearest five ^8, 60, 64^. Regrettably, zero is reported to be the actual number of Black physics professors ^8^. There are five Black professors reported in the biosciences out of 1,345 and around 20 Black engineering professors from 1,730. Overall, in all sciences, there were a total of 70 Black professors. This is just 0.6% of the number of science professors in the UK. Furthermore, of these 70, only 10 were Black women ^8^. A key challenge facing Black academics is that, whereas one in ten White and Asian academics can expect to be promoted to professors, the figure is one in twenty-five for Black academics, a figure that has not changed for at least 10 years and reflects the structural barriers in the system ^59^.

### The value of having Black professors

The use of role models is often suggested as a mechanism for increasing diversity in STEMM ^69^. STEMM role models can shape a student’s motivation by providing a positive example of success. The importance of seeing role models in a variety of spaces, including the education environment, at home, and on TV, cannot be emphasised enough ^70^, especially when facing a hostile environment due to “unconscious bias” or direct racism.

Role models have been shown to positively influence individuals’ decision making, and a systematic review of role models in STEM ^71, 72^ highlighted the importance of this. When considering role models, an overlooked impact is their effect on non-Black colleagues and students. This is equally important as by observing or directly working with a Black colleague or professor these gatekeepers gain new insights into the potential of Black colleagues and students they may have otherwise overlooked or misjudged when making decisions. In addition to, “ *you cannot be it, if you cannot see it*” ^73– 75^, just as important but often overlooked is: “ *you cannot be it, if the gatekeepers cannot see it”.*

### What is the STEMM pipeline

This search originally focused on Black Heritage researchers in biomedical research. However, owing to the research context, in terms of the number of Black heritage researchers and academics, this exploration has expanded to include Black heritage researchers from across STEMM.

The STEMM pipeline has been defined as the pathway to careers in STEMM from school to university ^76^. The Leaky pipeline is often described as the gradual and continuous loss of women at each career stage in STEMM from postdoctoral researchers to professors ^77, 78^. The Broken pipeline is thought to be the factors that ultimately contribute to the lack of Black academics in the UK ^28, 79^.

Failure to maintain the pipeline of Black students and researchers at its earliest stages ensures the exclusion of this group from the onset. Where they exist in the Higher Education system, they are generally concentrated in nonresearch-intensive institutions. Evidence suggests that Black students and researchers struggle to remain in the system and contribute to the development of UK Science and Technology, or progress at a speed and manner that is equal to their peers ^45, 56, 72^.

Black pupils have been recorded to constitute over 8% of all undergraduate admissions since 2015/16 ^45^, which is an overrepresentation of this group compared to the general population ^1, 61^. However, there are disparities in access to research-intensive universities among this minoritised group, which continue to be underrepresented in Russell Group universities. Black students were noted as 8.1% at post-1992 universities; nonetheless, they were remarkably only 3.3% at Russell Group universities ^6, 80^.

Unfortunately, the lack of academic career progression among Black students can be observed in the number of first-year entrants to postgraduate studies. In 2015/16, Black students accounted for 6.1% of these first-year entrants, which peaked at 7.8% the following year. There has been a steady decline in Black students, and the latest figures state that they are back to 6.6% of first-year postgraduate entrants ^45^. One issue with Black students and progression and completion in Higher Education Institutions is that many often do not complete the named degree they signed up for or take longer to complete, both of which have consequences for transition into postgraduate courses and later into employment.

### What is it about the research culture and environment in UK institutions that is preventing Black researchers from thriving?

The Royal Society states that research culture encompasses the behaviours, values, expectations, attitudes, and unwritten rules that dictate what is considered right, wrong, or acceptable behaviour, known as norms, within research communities, influencing how research is conducted, communicated, and how researchers are supported ^81^.

What does research culture mean from the perspective of researchers, especially Black researchers, and their ability to thrive? Within this framework, behaviour can be described as the process of conducting research. The factors that influence or steer how research is carried out, referred to as determinants of behaviour, consist of values, expectations, attitudes, and norms.

Thriving is often defined as a positive state of functioning comprising mental, physical, and social well-being. The tools and methods used to assess an individual's well-being and ability to flourish in various aspects of life range from self-report surveys to objective assessments of physical and social conditions ^82^.

In the above context, there are expectations from the researchers of themselves and the norms of research culture, such as: ‘Publish or perish’, repeatedly writing grants and funding applications, maintaining consistent and accurate high standards, and contributing towards teaching ^83^. Combining this with the expectations of the institute, leadership, or line management from researchers is vital to understanding the questions that arise from these perceptions. To illustrate, what do my line managers and departments expect of me? How do these expectations affect me? How do my managers’ attitudes towards me influence my actions? An additional challenge facing Black researchers is to navigate not only the conventional challenges facing aspiring researchers, but they must also meet the challenge of being and operating in a space that either does not recognise them, has low expectations of them, wants to exclude them, or does not see their contribution as valuable ^70^.

If research culture is about fostering a supportive, inclusive, and ethically sound environment in which researchers can thrive and contribute to high-quality research, then the collective negative expectations of people can lead to damaging impacts such as anxiety, deterioration in physical and mental health, and ultimately, delayed outputs ^41, 84^.

If this is the general academic culture, then how much more challenging is this environment for individuals, in this case Black researchers, in a system that historically has excluded them and who must work harder to dismantle the barriers and demonstrate to this system that they belong ^70, 84^. The theory of critical mass states that when a committed minority reaches a critical size, a cascade of behavioural changes can occur, overturning apparently stable social norms ^85^. Considering the low number of Black leaders in the Higher Education system, there is still a way to go before this critical mass is reached ^7^. In fact, isolated individuals in senior positions can give a false impression that the system is working and prevent change. This means that onus must sit with gatekeepers who control access to resources and opportunities ^86, 87^.

Following the re-establishment of the Black Lives Matter (BLM) movement in 2020, numerous articles in the media, newspapers, and academic journals led to heightened awareness of inequalities at all levels, from peers, managers, leadership, and the government ^5, 6, 86, 88– 93^. This awareness of inequalities and exclusion of minoritised groups, most notably Black heritage individuals, in all aspects of their being was seen on local, national, and international fronts. This scenario should be repeated within the academic research culture ^94^.

Much of the discussion and literature has been published in the United States, with limited accounts from the UK (Bhopal 2013). The “Beyond the data” report ^26^, identified the problem and made several practical recommendations about increasing the number of Black health and care professionals but again there has been little coordinated action in response to this report.

### Gatekeeping: systemic and structural inequalities

In view of several reports that highlight the stark and historic absence of data to enable meaningful discourse, the willingness of gatekeepers to objectively analyse and address the systemic drivers of underrepresentation and exclusion is urgently required ^6^.

The CRED report triggered criticism from several channels, owing to its minimisation of structural racism ^95, 96^. In their response to this report, the British Medical Association welcomed, *“this long-overdue focus on health inequalities as part of the wider conversation about race in the UK”* ^44^, but believes that *“attention should now be on implementation of solutions to address the known ethnic disparities and inequalities, with any further research focused on where it is required to support or monitor action.”* ^97^.

The systemic drivers of underrepresentation and exclusion include funding disparities ^98^, lack of transparency in funding processes, and lack of institutional support. Funding bodies also need to view applications and applicants from more diverse perspectives ^99^. In recent years, The Wellcome Trust has invested time and resources to improve research culture and inclusivity ^41^. Moreover, there have been ongoing efforts and funding from Research England, with funding for improving research culture ^87^ and medical school training, such as the BMA Charter against Racism ^100^. These initiatives are too few, account for a small fraction of the funding awarded each year, are often not sustainable beyond the life of the specific award, and are replaced by something unconnected.

In 2020 for the first time, the UK Research and Innovation (UKRI) published an analysis of ethnicity data for UKRI funding applicants and awardees ^101^. Updated data reported in 2021 ^102^ showed that while the proportion of fellowship awardees from minoritised ethnic groups had increased, fellows, specifically from the Black ethnic group, still constituted less than 1% of the awardees. They also account for less than 1% of Principle Investigators, which is well below the HESA estimate of academic and labour market share. UKRI admits not having full ethnicity information for UKRI-funded PhD studentships. Where data exist, minoritised ethnic groups account for 13% of the candidates, with the majority being Asian.

In 2021, the Head of the UKRI responded to the accusations in an open letter from ten Black women academics regarding the lack of transparency in funding processes ^103, 104^. The call was to address structural inequalities and disparities in COVID-19 outcomes in UK Black, Asian and Minority Ethnic communities. However, Black scientists were prevented from accessing these funds. The eventual response listed six areas in which UKRI would seek to address the concerns of Black academics regarding its funding processes and outcomes ^105^.

The Royal Society reported that minoritised groups are more likely than their peers to be unemployed six months after graduation compared to White STEM leavers ^56^ and to be on fixed-term (temporary) contracts, and they were also more likely to earn less than their peers at the same job level ^106– 108^. These are key obstacles to surviving and thriving in STEMM, particularly if you are from a Black or minoritised background, which means that you are more likely to be from a low socioeconomic group and are more impacted by low wages and job insecurity.

Moreover, some funding applications require institutional support to secure research funding, such as a percentage of matched contributions or costs towards large equipment for applicants. In this circumstance, institutions are more likely to fund senior academics who are also well connected; however, as Black researchers make up a tiny fraction of their senior academics and often do not have network connections ^109^, this disparity constitutes a significant attainment barrier.

### Evidence of Black researchers not thriving

Thriving can comprise life experiences and prospects for growth and prosperity ^110^. The structural and cultural characteristics of organisations influence the creation of radical innovations, which is a great case for inclusion in research, yet it is still a goal not often attained ^111^. We must now also consider the impact of governments in the USA and elsewhere, which are challenging the need for inclusion ^112, 113^. This is capable of creating a double hit on Black researchers, as it has long been reported that many Black academics have decided to emigrate to secure a meaningful career in the US academy rather than continuing their individual struggles in the UK ^114, 115^. There are numerous examples of Black researchers who have faced or discussed negative experiences and barriers to progress in academia ^7, 28, 33, 58, 70, 86, 116^. Another challenge for Black British researchers is the fact that a large proportion of Black researchers and academics currently based in the UK were born and started their educational journey overseas ^33^. This vacuum of Black UK-born and educated researchers requires vital scrutiny ^117– 119^. Additionally, there is evidence of structural barriers starting at the lowest levels of education and training and ineffective attempts to solve the problem by importing talent without the culture, processes, and investment to support Black talent.

During 2020, and the resurgence of the BLM movement, there was an elevated visibility of Black researchers and academics largely driven by necessity, and the realisation that specific support for Black researchers has been inadequate for many years, given the challenges they face underpinned by racialised bias.

There was increased local and worldwide community building and engagement with fellow Black scientist, helped by the use of hashtags like #BlackintheIvory #BlackinSTEM and the explosion of #Blackin“X” e.g. #BlackinCancer #BlackinNeuro etc ^120^. Making their own safe spaces to feel included in environments that had thus far excluded them was a necessary step, but not sustainable in terms of research careers, which requires wider cultural change.

While these are significant initiatives, in the short term, running these networks, which are often done by volunteers without additional resources invested, contributes to the phenomenon known as the “Minority Tax” ^121^. The Minority Tax has been described as the additional burdens and responsibilities placed on underrepresented individuals ^122, 123^. Consequently, despite being recognised and praised by institutions for their success, the added responsibilities cause an increased workload and can lead to reduced time for their primary duties, such as research and teaching, which rarely leads to benefits for individuals’ career prospects.

### What is it about the recruitment, retention and progression process in organisations that prevents successful Black researchers from gaining promotion?

Research careers are complex and diverse, and, in an increasingly competitive environment, skill development and the creation of professional networks are key contributors to progression. Achieving a research degree from a research-intensive institution and other accomplishments plays a principal role in recruitment in the early stages, and is essential for progression into senior academic roles ^124^. Research outputs are widely recognised as the key to academic success, with a huge emphasis on the ‘publish or perish’ narrative with a specific focus on publication output ^125^. In addition to teaching obligations, senior academics need to be equipped with skills and experience in academic management, fundraising, curriculum planning, supervision, outreach activities, and more.

Evidence documenting the lived experiences of 20 (out of 25) Black female professors ^33^ identified several barriers to career progression, including disproportionate workloads compared to other colleagues and lack of clarity and transparency in processes that guide recruitment and progression. It could be concluded that Black researchers are more likely to be kept busy with tasks that did not provide the skills and networks needed to progress and were overworked to the point of not prioritising or expanding their professional portfolio ^124^.

In addition, Black female professors experienced bullying and racial harassment as well as direct and indirect discrimination. Adding to these factors that can affect progression and promotion in academia, is the high level of job insecurity within Higher Education Institutions, particularly at the lower levels where most Black researchers spend most of their careers. This job insecurity affects their ability to publish, develop independent research profiles, and form effective collaborative connections ^126, 127^.

This experience is not limited to biomedical researchers and academics. In the article ‘Missing Elements: Racial and ethnic inequalities in the chemical sciences’, it states that Black chemists leave the profession at every stage of their career path after undergraduate studies ^64^. The promotion and progression of Black and ethnically minoritised teachers in England has been the subject of much debate. Although several theories have been proposed, a lack of racial equity has emerged as a major contributing factor. The experience of teachers within the profession points to similar patterns of exclusion ^128^. Similarly, Black engineers in the UK motorsport industry interviewed by The Hamilton Commission expressed a sense of frustration in the lack of progression through to leadership roles, highlighting microaggressions and racist banter that they were compelled to “put up with” ^129^. These experiences and the general lack of commitment from industry leadership likely contribute to the exodus of the limited talent that exists in the industry.

Regarding the healthcare workforce, the “snowy white peaks” of the NHS survey on discrimination in governance and leadership and the potential impact on patient care in London and England ^130^ showed that there remains a significant gap between the composition of Trust Boards, national NHS bodies, and the rest of the workforce and the local population. Evidence suggests that this may adversely impact the provision of services, denying the NHS the potential contribution of diverse leadership ^131^. The rate of change in the NHS towards racial equity has been glacial ^132, 133^, and a comparable study at this level among the academic researcher workforce has not been conducted. It is likely that a similar situation exists, with similar effects on research output and real-world impacts.

The evidence demonstrates that Black people experience similar barriers with comparable negative outcomes, culminating in underrepresentation at the senior level in many industries, not just healthcare and academia. It is, therefore, a matter of urgency to design and enact evidence-based interventions to address the low number of Black academics in the UK academic and research system. One obvious starting point is the leadership of research commissioning bodies that drives the research agenda, determining how questions are framed, what data inform them, and how patients and the public are involved ^134– 137^ *.* Addressing inequalities in research careers, including biomedical research, is timely and the only way to ensure that we see real and sustainable research impacts.

## Conclusions

This paper places a spotlight on the research culture in UK institutions, which prevents Black researchers from thriving and confirms the need for real policy changes, underpinned by an anti-racist approach to the implementation of many recommendations from numerous studies over the last 40 years.

Initiatives that foster connections, demonstrate resilience, and create a sense of belonging among young Black researchers also contribute to the uncompensated labour on diversity efforts that limit time for research and hinder career advancement. Noticeably, the underrepresentation of Black professors is not solely a pipeline issue but the outcome of structural inequalities in funding, recruitment, and workplace culture. The absence of Black professionals in senior roles is evidence of an ethos of cultural exclusion and systemic bias rather than individual inadequacy. This absence is observed in biomedical research, Higher Education Institutions, and across sectors.

These issues intersect to prevent Black researchers and academics from thriving, and their absence has broader implications: lack of representation affects student aspirations, reinforces racial bias, and limits intellectual diversity in research and teaching.

Addressing these disparities requires urgent and systemic interventions. Research bodies and institutions must adopt an antiracist approach to funding equity, transparent recruitment and promotion, and accountable leadership.

***"The beauty of anti-racism is that you don't have to pretend to be free of racism to be an anti-racist. Anti-racism is the commitment to fight racism wherever you find it, including in yourself and it's the only way forward."***
                                                                                                                                                                                                                                                           
***Ijeoma Oluo 2019***

## Ethics and consent

Ethical approval and consent were not required.

## Data Availability

No data are associated with this article.
