# Supplementary material for: Where are my Black professors? The research culture preventing Black researchers from thriving in UK institutions
Source: Wellcome Open Res. 2025 Sep 4;10:480. [Version 1] doi: 10.12688/wellcomeopenres.24584.1 (PMC12949837; doi:10.12688/wellcomeopenres.24584.1)
Supplement: Peer review history

# Reviewer report: Reviewer response for version 1

**DOI:** 10.21956/wellcomeopenres.27093.r146894

**Published:** 2026-02-28

**Authors:** Eghosa Obaizamomwan-Hamilton

**Affiliations:** 1 Stanford University, Stanford, CA, USA

This work articulates the many barriers that get in the way of Black scholars. Things like gatekeeping and structural obstacles are increasing ethnic disparities in the UK. Very interested in the "vacuum" of Black researchers over the years and the role of the minority tax on Black researchers. There are strong implications for the recruitment and retention of Black scholars and the underrepresentation in senior levels.

Is the review written in accessible language?

Yes

Are all factual statements correct and adequately supported by citations?

Yes

Are the conclusions drawn appropriate in the context of the current research literature?

Yes

Is the topic of the review discussed comprehensively in the context of the current literature?

Yes

Reviewer Expertise:

Sociology of Race and Education, Black Educational Studies, Black Feminist Thought, Endarkened Feminist Epistemology, Intersectionality, Critical Pedagogy, Black Critical Race Theory, Women’s Studies

I confirm that I have read this submission and believe that I have an appropriate level of expertise to confirm that it is of an acceptable scientific standard.

# Reviewer report: Reviewer response for version 1

**DOI:** 10.21956/wellcomeopenres.27093.r133637

**Published:** 2025-10-16

**Authors:** Monica L. Miles

**Affiliations:** 1 University at Buffalo, Buffalo, USA

Reading “Where Are My Black Professors?” by Essilfie-Quaye et al. felt like encountering a deeply familiar truth—one that many Black academics like myself live daily, yet rarely see so thoroughly documented. The authors have crafted a powerful and necessary critique of the UK’s academic research culture, exposing the systemic and structural barriers that prevent Black researchers from thriving. Their framing of racism, not as isolated incidents but as embedded within institutional norms, funding structures, and cultural expectations, is both bold and essential.

 The paper’s strength lies in its thematic clarity. By organizing the review around representation, research culture, recruitment, and gatekeeping, the authors provide a comprehensive map of exclusion. The discussion of the “Minority Tax,” the broken STEMM pipeline, and the invisibility of Black professors in elite institutions resonates deeply and offers readers a sobering view of the landscape.

 However, I found myself wanting more clarity around the methodological framing. While presented as a literature review, it would benefit from explicitly stating whether it is a scoping review, narrative review, or another form. Including a brief description of how sources were selected and how themes were derived would help readers understand the analytical process and enhance scholarly rigor.

 I also believe the paper could be enriched by drawing on comparative frameworks, particularly from the U.S. For example, Ebony McGee’s work on “racial battle fatigue” and the emotional toll of navigating elite spaces as a Black scholar adds depth to the discussion of thriving versus surviving.

 Two U.S.-based studies may be especially useful:

 Miles, M. L., McGee, E. O., & Botchway, P. (2024). Mixed reviews on diversity initiatives: toward an institutional change model for Black faculty in engineering and computing. Frontiers in Education, 9, 1324389. https://doi.org/10.3389/feduc.2024.1324389

 This study identifies stagnant, moderate, and transformational institutional environments and offers a framework for moving beyond performative diversity efforts.

 Miles, M. L., Morton, T. R., Pettis, P., Henderson, R., Frizell, C. A., Bester, V. S., ... & Trevino-Fica, L. (2025). Navigating racism: A scoping review of barriers in the experiences of Black Health Professions students. Discover Education, 4(1), Article 153. https://doi.org/10.1007/s44217-025-00558-8

 This review categorizes barriers across interpersonal, institutional, and systemic levels and calls for policy responses to dismantle structural racism.

 Though U.S.-based, these works highlight structural parallels that transcend national boundaries. Including them would broaden the scope and offer readers a richer understanding of institutional racism.

 Finally, while the paper focuses on exclusion, I encourage the authors to also name rare instances of transformational environments, spaces where Black faculty feel supported and empowered. These examples, though few, offer a vision of what is possible and can serve as aspirational models for institutional change.

Is the review written in accessible language?

Yes

Are all factual statements correct and adequately supported by citations?

Yes

Are the conclusions drawn appropriate in the context of the current research literature?

Yes

Is the topic of the review discussed comprehensively in the context of the current literature?

Partly

Reviewer Expertise:

Literature review on racism and Black faculty in STEMM

I confirm that I have read this submission and believe that I have an appropriate level of expertise to confirm that it is of an acceptable scientific standard.

## References

[1] : Mixed reviews on diversity initiatives: toward an institutional change model for Black faculty in engineering and computing. *Frontiers in Education* .2024;9: 10.3389/feduc.2024.1324389 10.3389/feduc.2024.1324389

[2] : Navigating racism: A scoping review of barriers in the experiences of Black Health Professions students. *Discover Education* .2025;4(1) : 10.1007/s44217-025-00558-8 10.1007/s44217-025-00558-8
